# The contribution of microglia to early synaptic compensatory responses that precede β-amyloid-induced neuronal death

**DOI:** 10.1038/s41598-018-25453-1

**Published:** 2018-05-08

**Authors:** Sara Merlo, Simona Federica Spampinato, Martina Beneventano, Maria Angela Sortino

**Affiliations:** Department of Biomedical and Biotechnological Sciences, section of Pharmacology, University of Catania, Catania, Italy

## Abstract

Glial-neuronal cross-talk has a critical role in the development of neurodegenerative conditions, including Alzheimer’s Disease, where it affects neuronal responses to β-amyloid peptide (Aβ)-induced toxicity. We set out to identify factors regulating synaptic responses to Aβ, dissecting the specific role of glial signaling. A low concentration of aggregated Aβ42 induced selective up-regulation of mature brain-derived neurotrophic factor (BDNF) expression and release in rat organotypic hippocampal cultures as well as in cortical pure microglia. Conditioned media from resting (CMC) or Aβ42-treated (CMA) microglia were tested for their effects on synaptophysin expression in SH-SY5Y neuronal-like cells during challenge with Aβ42. Both CMC and CMA prevented Aβ-induced synaptophysin loss. In the presence of Aβ + CMA, synaptophysin was over-expressed, although it appeared partly clumped in cell bodies. Synaptophysin over-expression was not directly dependent on BDNF signaling on neuronal-like cells, but relied on autocrine BDNF action on microglia. FM1-43 labeling experiments revealed compromised synaptic vesicle recycling in Aβ42-treated neuronal-like cells, rescued by microglial conditioned medium. In these conditions, significant and prolonged neuroprotection was observed. Our results point to microglia as a target for early intervention, given its positive role in supporting neuronal compensatory responses to Aβ synaptotoxicity, which potentially lead to their extended survival.

## Introduction

Alzheimer’s Disease (AD) is a progressive neurodegenerative condition affecting millions worldwide, an incidence destined to increase along with population ageing^1,2^. To date, the multifaceted pathophysiology of AD is not yet fully understood and effective therapeutic options are lacking^3^. The central event in AD onset appears to be the abnormal accumulation of the 42 residue-long form of beta-amyloid peptide (Aβ42) ultimately making of AD a protein misfolding disease^4^. Aβ42 is characterized by a high rate of aggregation and self-assembles in progressively higher molecular weight structures^1,3^. Oligomeric Aβ has been identified as the most toxic species, responsible for early cell responses which trigger synaptic damage and consequent neuronal death^5–7^. In more advanced stages of the disease, accumulation of extracellular deposits of Aβ fibrils leads to plaque formation, chronic inflammation and massive neuronal death, clinically coinciding with a progressively severe cognitive decline^1^. Aβ42 accumulation is recognized to exert a primary role in the progression that eventually culminates in neuronal death, but in parallel, formation of neurofibrillary tangles occurs, characterized by intraneuronal aggregates of hyperphosphorylated microtubule-associated protein *tau*^8^.

In neurodegenerative diseases, neuronal degeneration is profoundly affected by the action of glial cells^9^. Microglia and astrocytes are swiftly activated by exposure to toxic stimuli and give rise to a complex signaling interplay which involves both autocrine and paracrine effects^10–12^. Among the many effectors released by glial cells is brain-derived neurotrophic factor (BDNF), a neurotrophin characterized in AD by a biphasic pattern of expression with a significant rise in patients showing mild cognitive impairment (MCI), followed by a substantial drop in patients at more advanced stages of the disease, when neuritic degeneration and reduced spine density occur^13^. Notably, BDNF from microglia has been shown to display both a pro-inflammatory autocrine action^14^ and a role in synaptic formation and function *in vitro*^15^.

In our recent work using organotypic hippocampal cultures (OHC), we showed that chronic exposure to low concentrations of Aβ, a condition of slow-developing neuronal damage, triggered an early response involving increased expression of pre-synaptic protein synaptophysin (SYP) and enhanced synaptic activity^16^. In this model, initial SYP increase was interpreted as an attempted compensatory response to synaptic failure, and was indeed followed by SYP reduction at later time points, when neuronal death also occurred^16^. The analogy between the biphasic pattern of expression we observed for SYP and that reported in literature for BDNF, led us to hypothesize the existence of a link between BDNF and SYP over-expression. We thus asked whether BDNF could be the mediator of an early survival response to Aβ toxicity, promoting synaptic activity through SYP increase. In addition, we explored the role of BDNF of glial origin, together with pro-inflammatory cytokine tumor necrosis factor-α (TNF-α), as possible mediators of glia-neuron crosstalk in this context. To this end, we used organotypic hippocampal slices and individually cultured neuronal-like cells, astrocytes and microglia.

## Results

### BDNF expression is increased early during exposure to Aβ42 in OHC and pure microglia

**Figure 1:**
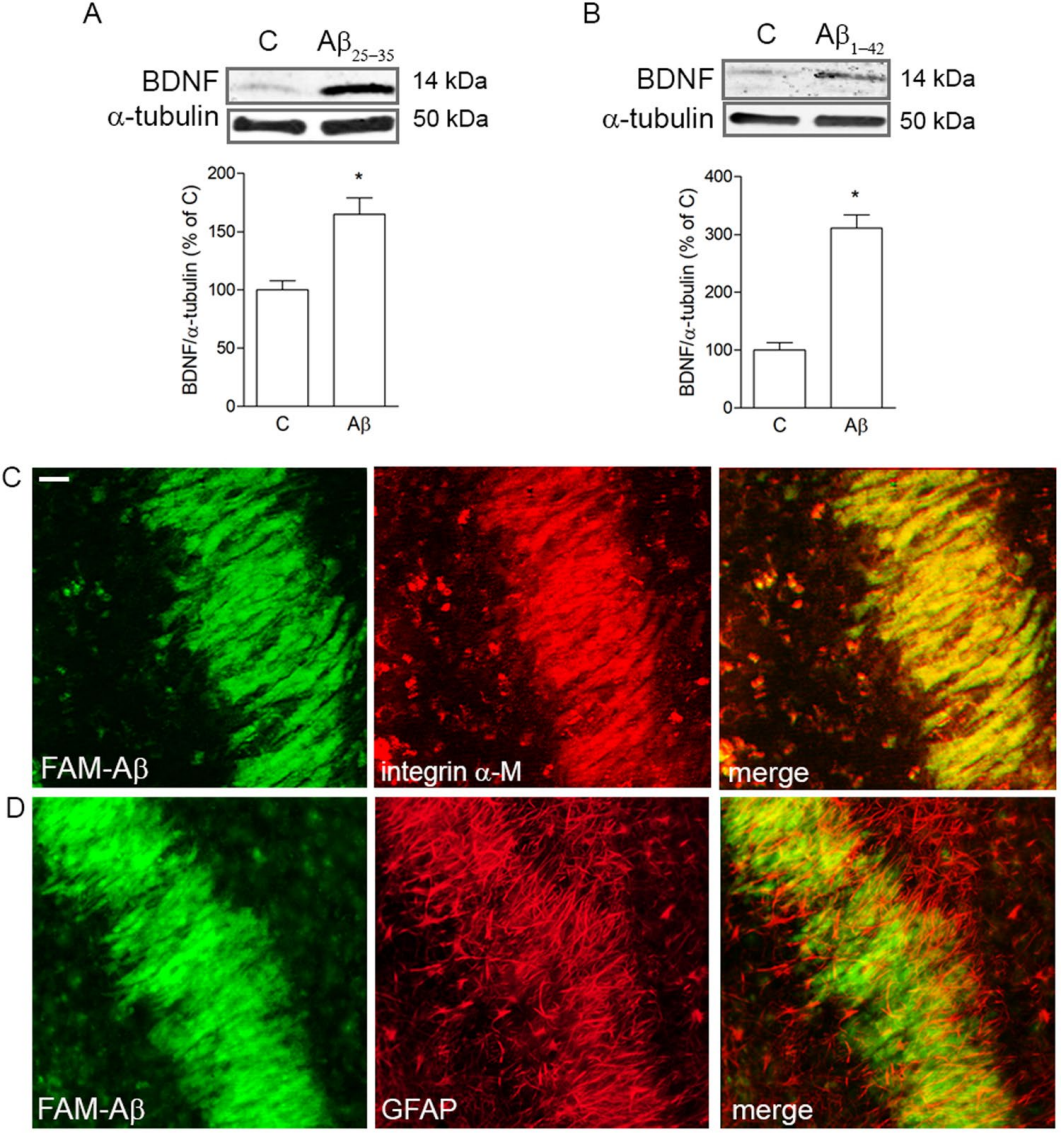
Aβ up-regulates mature BDNF expression and co-localizes with microglia in OHC. Western blot analysis of BDNF expression in OHC slices treated with 2 μM Aβ25-35 for 24 h (**A**) or 0.5 μM Aβ42 for 7 days (**B**). Co-localization of FAM-Aβ (green) in CA1 area of OHCs with cell type markers, integrin α-M (red, **C**) and GFAP (red, **D**), observed 24 h after its addition to the slices. Scale bar is 20 μm. Values are mean ± SEM with n = 3. *p < 0.05 vs. control by Student’s t-test.

BDNF expression was evaluated in OHC exposed to sub-lethal concentrations of either Aβ25-35 (2 μM for 24 h) or Aβ42 enriched in oligomers by aggregation (0.5 μM for 7 d). These are conditions of slow developing neuronal damage that, as previously shown by our group, coincide with Aβ-driven SYP increase at a time when neuronal death is still negligible^16^. Western blot analysis showed a significant rise in mature BDNF expression (14 kDa) after treatment with both Aβ25-35 (Fig. 1A) and Aβ42 (Fig. 1B). Notably, when fluorescently labeled FAM-Aβ42 (0.25 μM) was applied to OHC for 24 h, it displayed a strong tropism towards integrin α-M-positive microglia (Fig. 1C) yielding a superimposable staining. Microglial cells appeared elongated and regularly aligned. No co-localization was evident between FAM-Aβ42 and GFAP-positive astrocytes (Fig. 1D), although they appeared similarly aligned. Neuronal morphology in the area was analyzed by staining with selective marker MAP2 (Supplementary Fig. 1).

**Figure 2:**
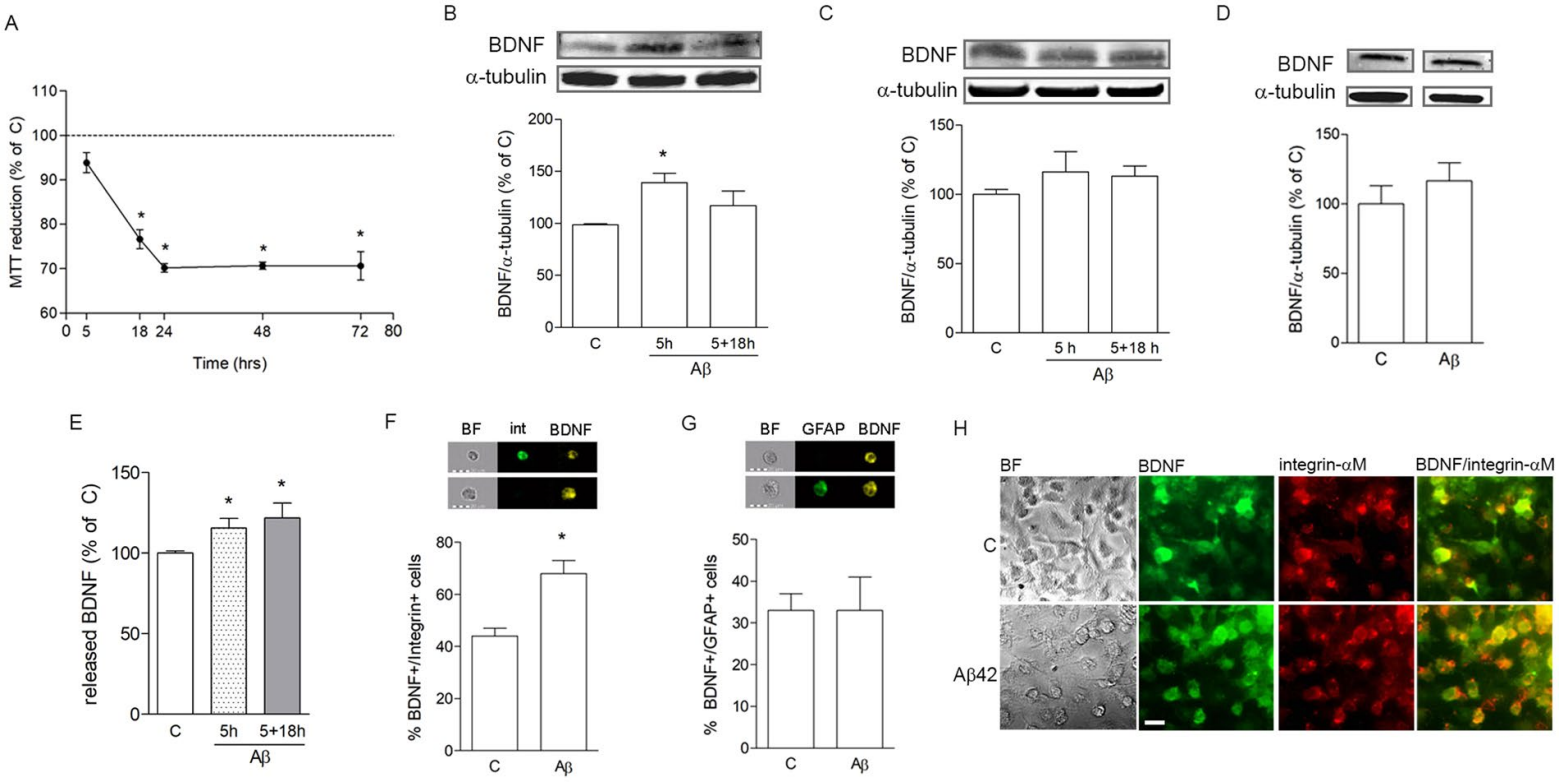
Aβ42 selectively induces microglial mature BDNF expression and release. MTT analysis of Aβ42 (0.5 μM) toxicity on differentiated SH-SY5Y cells treated for different time points, as indicated (**A**). Western blot analysis of BDNF expression in pMG (**B**) or pAS (**C**) treated with Aβ42 (0.5 μM) for 5 h or pulsed for 5 h followed by medium change and further incubation without Aβ for 18 h (labeled as 5 + 18 h). Western blot analysis of BDNF expression in SH-SY5Y cells exposed to Aβ42 (0.5 μM) for 5 h (**D**). ELISA determination of released BDNF in medium collected from pMG treated for 5 h or 5 + 18 h (**E**). Image cytofluorometric analysis of co-localization of BDNF with integrin α-M (int; **F**) or GFAP (**G**) in mxG cells. BF = brightfield. Double immunostaining of BDNF (green) and integrin α-M (red) in control vs Aβ42-treated (0.5 μM for 5 h) pMG (**H**). Scale bar is 20 μm. Blots were cropped to display specific bands. Original blots are reported in Supplementary Fig. 1. Values are mean ± SEM with n = 3-5. *p < 0.05 vs. untreated control by one-way ANOVA followed by Newman-Keuls test for significance or Student’s t-test, as appropriate.

Astrocytes, microglia and neuronal-like cells were separately cultured to test individual effects of Aβ42 on BDNF expression and downstream effects. Primary rat cortical astrocytes and microglia were either grown as a mixed culture (mxG) or separated and cultured as pure astrocytes (pAS) and pure microglia (pMG). Neuroblastoma SH-SY5Y cells were subjected to gradual serum reduction in order to arrest cell cycle and induce neuronal differentiation. Treatments were always carried out in serum-free conditions (SF). Reduction of neuronal-like cell proliferation in these conditions was confirmed by cytofluorometric analysis of cell cycle distribution following propidium iodide incorporation. Time-course analysis of Aβ42 toxicity at 0.5 μM was performed by the MTT assay on neuronal-like cells and showed significant cell death starting at 18 h, with maximal effect at 24 h of exposure (Fig. 2A). Based on this result, 5 h was chosen as an early time point preceding cell death in order to analyze synaptic protein content in SH-SY5Y cells, while effects of treatments of interest on neuronal viability were tested at 24 h or longer. MTT assay revealed no Aβ42-induced cell death for pMG or pAS in the same conditions (not shown).

Expression of the mature form of intracellular BDNF (14 kDa) was selectively analyzed, and in our Western blot and ELISA results we will always refer to this specific form. Western blot analysis of intracellular BDNF content was significantly increased in pMG after 5 h of exposure to 0.5 μM of Aβ42 (Fig. 2B). When pMG were pulsed for 5 h with Aβ42, followed by Aβ removal and an 18 h-recovery (5 + 18 h; Fig. 2B), there was still a trend towards BDNF increase, which however was less pronounced and did not reach statistical significance (Fig. 2B). At the contrary, treatment with Aβ42 did not significantly modify BDNF expression in pAS after 5 h or 5 + 18 h (Fig. 2C), nor in differentiated SH-SY5Y cells after 5 h (Fig. 2D). Consistent with cell content expression results, ELISA measurement of BDNF in Aβ-treated pMG-derived conditioned medium (CM) showed a significant increase after 5 h of exposure to 0.5 μM of Aβ42 but also after the pulse/removal treatment as described above (Fig. 2E). To further confirm selectivity of Aβ42 for stimulation of microglial BDNF expression, imaging flow cytometry experiments were carried out using mixed glial cultures (mxG). Cells were immunostained for BDNF (yellow, Fig. 2F) coupled to either microglial marker integrin α-M (green, Fig. 2F) or astrocyte marker GFAP (green, Fig. 2G) in double staining experiments. The percentage of microglial cells co-expressing BDNF/integrin α-M significantly increased following Aβ42 treatment (0.5 μM for 5 h) compared to control (Fig. 2F). At the contrary, the percentage of GFAP+ astrocytes expressing BDNF was unaffected by exposure to Aβ42 (0.5 μM for 5 h; Fig. 2G). Figure 2H shows integrin α-M-positive pMG (red) co-immunostained with BDNF (green) in control and Aβ42-treatment conditions (0.5 μM for 5 h), the latter showing more widespread and intense staining for BDNF. Overall, these data point to microglial cells as early responders to Aβ42 exposure, presenting with increased expression and release of mature BDNF.

### Factors of microglial origin modify SYP expression in the presence of Aβ42

To evaluate how the presence of microglial medium, enriched in mature BDNF following treatment with Aβ, affected neuronal response to direct Aβ exposure, an appropriate experimental protocol was designed to avoid carry-over of Aβ from microglial treatment. As previously mentioned, in all experiments pMG were pulsed with 0.5 μM Aβ for 5 h, washed and let recover for 18 h to obtain final CM devoid of Aβ, to be transferred onto neuronal-like cells. CM was always serum-free. SH-SY5Y cells were challenged with Aβ42 in this pMG-derived CM from untreated (CMC) or Aβ-treated (CMA) conditions to examine its effects on synaptic protein levels and on neuronal viability.

**Figure 3:**
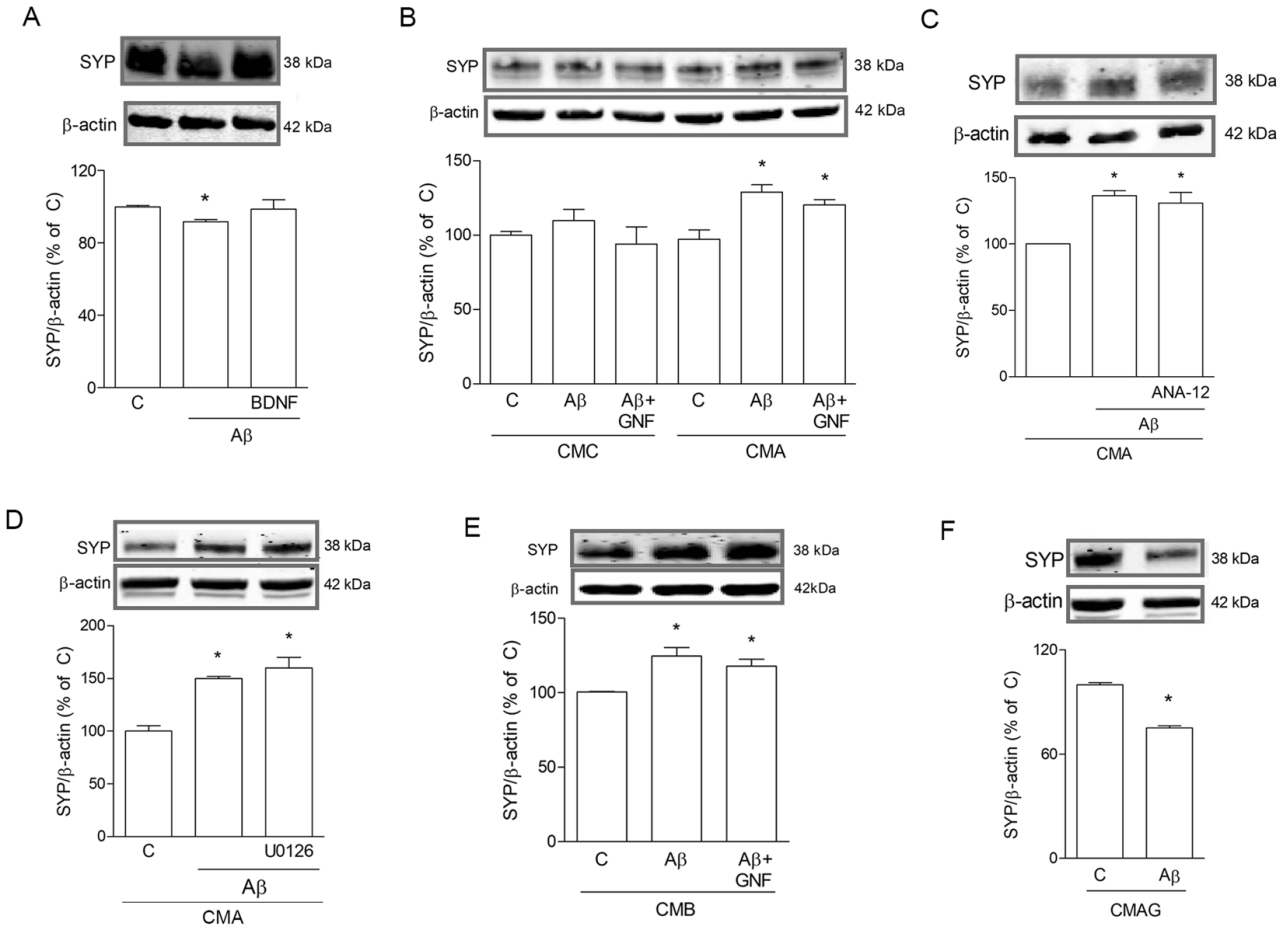
CM from microglia prevents Aβ42-driven SYP loss. Western blot analysis of SYP in SH-SY5Y cells exposed for 5 h to Aβ42 (0.5 μM) in different conditions. In A, cells were treated with Aβ42 alone or in combination with BDNF in non-conditioned medium (**A**). In (**B**), cells were exposed to Aβ42 alone or in combination with GNF5837 (GNF) in CM from untreated pMG (CMC) or from pMG treated with Aβ according to the 5 + 18 h-pulse protocol (CMA). Cells were treated with Aβ42 alone or in combination with ANA-12 (20 μM; **C**) or U0126 (10 μM; **D**) in the presence of CMA. Cells were treated with Aβ42 alone or in combination with GNF (100 nM) in CM from BDNF (1 ng/ml)-treated pMG (CMB; **E**) or CM from Aβ42 + GNF-treated pMG (CMAG; **F**). Blots were cropped to display specific bands. Original blots are reported in Supplementary Fig. 2. Values are mean ± SEM with n = 3–4. *p < 0.05 vs. respective controls by one-way ANOVA followed by Newman-Keuls test for significance or Student’s t-test, as appropriate.

Western blot analysis of SYP expression in SH-SY5Y cells was tested after exposure to 0.5 μM of Aβ42 for 5 h. In the presence of non-conditioned medium (NCM), Aβ42 produced a small, yet significant reduction of SYP content compared to control (Fig. 3A). Addition of BDNF (1 ng/ml) during Aβ42 exposure contrasted such reduction (Fig. 3A). Thus, exogenous BDNF, in the absence of additional factors, was able to prevent synaptic damage appearing early after exposure to Aβ42. When SH-SY5Y cells were treated with Aβ42 (0.5 μM for 5 h) in CMC, SYP protein levels were not anymore reduced compared to control condition (CMC alone; Fig. 3B). This effect was preserved when pan-Trk receptor inhibitor GNF5837 (100 nM; GNF) was present with Aβ42 (Fig. 3B). At the contrary, CMA not only prevented, but reversed the effect of Aβ42 treatment on neuronal-like cells, by significantly increasing SYP content (Fig. 3B). Such effect was again not modified by GNF (Fig. 3B), nor by the selective TrkB antagonist ANA-12^17^ (20 μM; Fig. 3C). As shown in Supplementary Fig. 4, ANA-12 was effective in blocking exogenous BDNF action. When the MAPK pathway, classically induced by BDNF^18^, was inhibited with U0126 (10 μM), Aβ42-induced SYP increase in CMA was not prevented (Fig. 3C). These results highlight the protective role of CM from both resting and Aβ42-stimulated microglia, while evidencing how specific factors released in the latter condition are responsible for distinct effects on synaptic proteins. Surprisingly, these did not depend on direct action of microglial mature BDNF on neurons. On the other hand, autocrine BDNF action on microglia proved to be essential in CMA action. In fact, CM yielded by direct treatment of pMG with BDNF (1 ng/ml for 24 h; CMB), was able to fully mimic the effects shown for CMA (Fig. 3D). This is consistent with CMB carrying, like CMA, BDNF and its paracrine-induced microglial factors. Moreover, the medium resulting from incubation of pMG with Aβ42 plus 100 nM GNF maintained during both Aβ pulse and subsequent recovery (CMAG), completely lost the ability to contrast Aβ42 effects on SYP expression (Fig. 3E). This is in line with a condition where Aβ42-induced rise in BDNF is not able to evoke further responses by microglial cells, due to receptor inhibition.

**Figure 4:**
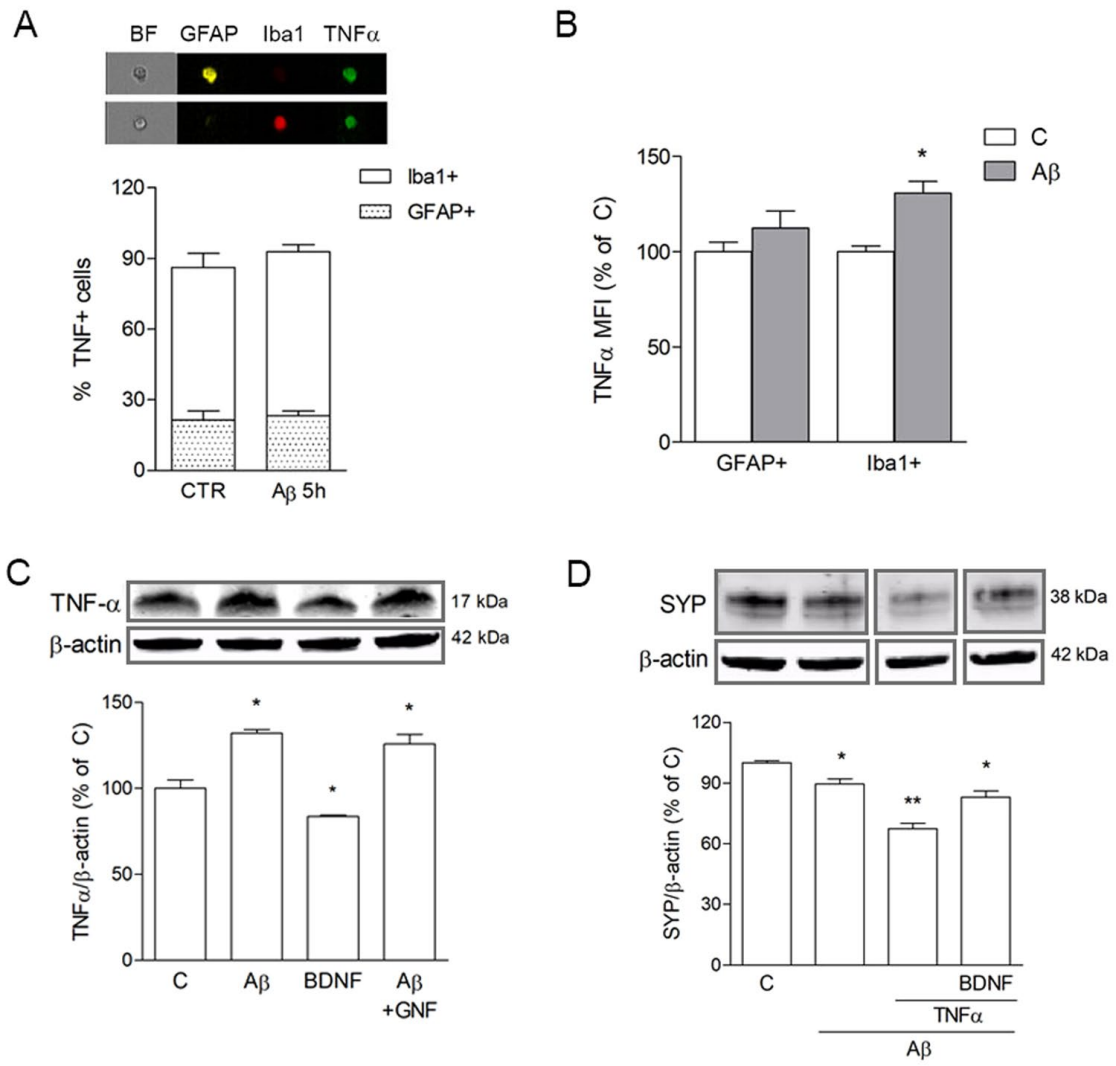
Microglial TNF-α is induced by Aβ42 independently of BDNF. pMG were treated with Aβ42 (0.5 μM) for 5 h. Co-localization of TNF-α with Iba1 or GFAP in mxG cells was determined by triple immunostaining followed by image cytofluorometric analysis of the percentage of immunopositive cells (**A**) and mean fluorescence intensity of TNF-α (MFI; **B**). BF = brightfield. Western blot analysis of TNF-α in pMG treated with BDNF (1 ng/ml for 24 h) or with Aβ42 (0.5 μM for 5 h) alone or in combination GNF (100 nM; **C**). Western blot analysis of SYP in SH-SY5Y cells treated with Aβ42 (0.5 μM for 5 h) alone or in combination with TNF-α (10 ng/ml) and BDNF (1 ng/ml) (**D**). Blots were cropped to display specific bands. Original blots are reported in Supplementary Fig. 3. Values are mean ± SEM with n = 3–5. *p < 0.05 vs. untreated control by one-way ANOVA followed by Newman-Keuls test for significance.

TNFα was tested as a potential candidate for microglial autocrine action of BDNF, based on recent literature^14^. We hypothesized that the presence of TNF-α in CMA could balance out the beneficial action of BDNF, accounting for the exceeding rise of synaptic proteins observed. Triple staining analysis of TNF-α co-expression with either GFAP-positive astrocytes, or Iba1-positive microglial cells, was carried out on mxG samples by imaging flow cytometry. Results showed that exposure to Aβ42 (0.5 μM for 5 + 18 h) significantly increased mean fluorescence intensity of TNF-α immunostaining (Fig. 4A), without affecting the percentage of positive cells (Fig. 4B), selectively in Iba1 co-labeled microglia. As shown in Fig. 4C, Western blot analysis of TNF-α expression in pMG confirmed a significant increase of TNF-α content following Aβ42 exposure (0.5 μM for 5 + 18 h), although such effect was not prevented by GNF (100 nM), nor mimicked by direct BDNF treatment (1 ng/ml). Interestingly, when directly added to SH-SY5Y cells, TNF-α (10 ng/ml) significantly enhanced Aβ-driven SYP reduction, and this effect was attenuated by direct addition of BDNF (1 ng/ml; Fig. 4D). These results confirm a detrimental role for TNF-α, but rule out its connection to BDNF actions.

### Microglial CM rescues synaptic function compromised by Aβ42

**Figure 5:**
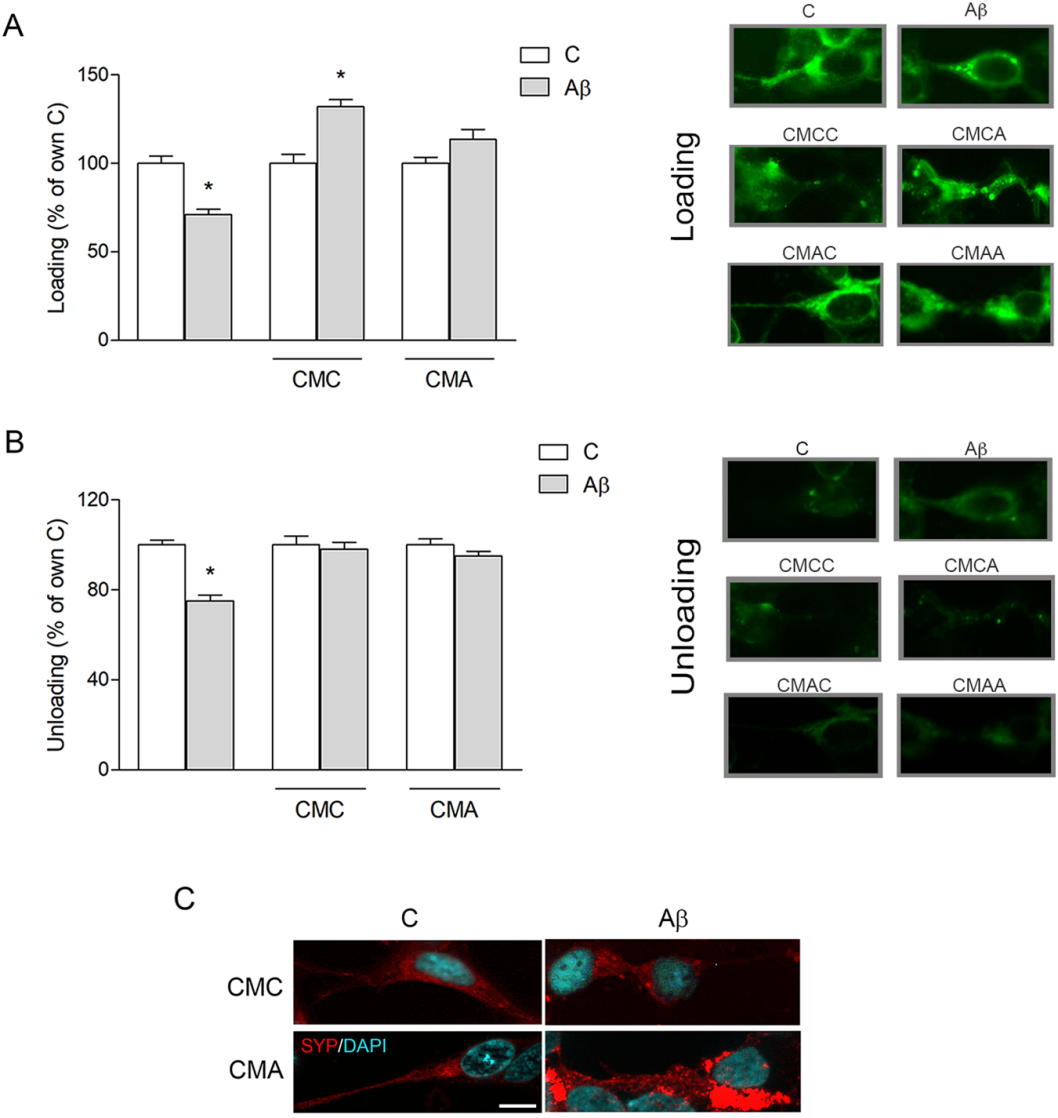
CM from microglia restores synaptic activity impaired following Aβ42 exposure. SH-SY5Y cells were treated for 5 h with Aβ42 (0.5 μM) in non-conditioned medium or in CM from untreated (CMC) or Aβ-treated (CMA) pMG. Cells were stained with FM 1–43 dye and treated to allow formation of labeled synaptic vesicles (loading). Fluorescence intensity was evaluated at the end of the loading step (**A**) and after unloading of fluorescent vesicles by stimulation of exocytosis (**B**). Representative images of cells stained with FM 1–43 in each condition, in the loading and unloading step, are reported next to corresponding graphs. Immunostaining of SYP (red) in SH-SY5Y cells exposed to Aβ42 (0.5 μM) for 5 h in CMA (**C**). Nuclei are stained with DAPI (blue). Values are mean ± SEM with n = 3–5. Scale bar is 10 μm. *p < 0.05 vs. respective controls by one-way ANOVA followed by Newman-Keuls test for significance.

Synaptic activity in different treatment conditions was determined by FM 1–43 dye labeling on SH-SY5Y cells. Aβ treatment (0.5 μM for 5 h) significantly reduced recycling of synaptic vesicles (Fig. 5A), measured as intensity of fluorescence at the end of the *loading* step (see Materials and Methods section). This effect was prevented in the presence of CMA, and reversed in CMC, where Aβ induced a significant increase in vesicle recycling compared to CMC alone (Fig. 5A). Release of synaptic vesicles, measured as percentage of fluorescence destaining induced by KCl stimulation, was likewise reduced by Aβ in basal conditions but completely restored in the presence of both CMC and CMA vs respective controls (Fig. 5B). Representative images of cellular FM 1–43 staining, in each condition, are shown next to loading (Fig. 5A) and unloading (Fig. 5B) graphs. SYP immunostaining of SH-SY5Y cells in the presence of CMA showed an even distribution of uniformly-sized synaptic puncta along an axon-like extension (Fig. 5C), as opposed to bright over-saturated clusters of SYP accumulating near the cell nucleus in the presence of CMA + Aβ (5 h; Fig. 5C). These results point to overexpression of SYP as the response to an aberrant accumulation of dysfunctional protein in the cell body. Data also suggest that this mechanism succeeds in compensating the partial lack of function, restoring vesicle release levels.

### Microglial CM enhances neuronal resistance against Aβ42 toxicity

**Figure 6:**
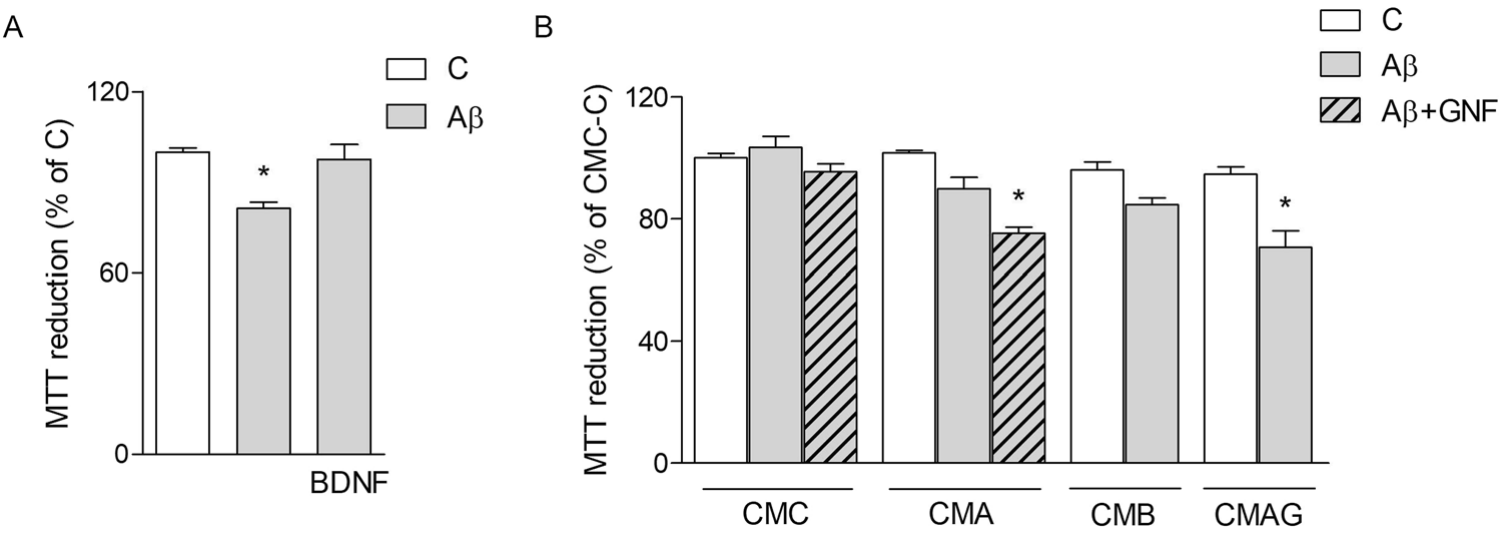
CM from microglia delays Aβ42-induced neuronal death. MTT assay on SH-SY5Y treated with Aβ42 alone or in combination with BDNF (1 ng/ml) or GNF (100 nM) in non-conditioned medium (**A**) and in CM from untreated (CMC), Aβ-treated (5 + 18 h; CMA), BDNF-treated (CMB) or Aβ + GNF-treated (CMAG) pMG (**B**). Values are mean ± SEM with n = 5. *p < 0.05 vs. respective controls. **p < 0.05 vs CMA + Aβ. Data were analyzed by one-way ANOVA followed by Newman-Keuls test for significance.

Finally, viability was determined by MTT assays performed on SH-SY5Y cells challenged with 0.5 μM of Aβ42 for 24 h either in non-conditioned medium or in the presence of CMC or CMA. In basal conditions, BDNF (1 ng/ml) prevented Aβ toxicity (Fig. 6A). Both CMC and CMA were able to contrast Aβ42 toxicity (Fig. 6B). This result suggests that prevention of SYP loss indeed correlates with enhanced survival in the presence of microglial CMs. Blockade of BDNF signaling in neuronal-like cells by addition of pan-Trk inhibitor GNF (100 nM) restored Aβ toxicity in CMA, but not in CMC (Fig. 4). Likewise, conditioned medium from pMG treated with BDNF (1 ng/ml; CMB) mimicked CMA effects by reducing Aβ toxicity on neuronal-like cells (Fig. 6B). Finally, CM derived from pMG exposed to Aβ42 under blockade of BDNF signaling with GNF (100 nM; CMAG) reinstated full Aβ toxicity on neuronal-like cells. Overall these data confirm the role of Aβ42-induced microglial BDNF in an attempt to increase cell survival.

## Discussion

The main purpose of the present study was to pinpoint new mechanisms of neuroprotection that come into play early during slow-developing, Aβ-induced, neuronal damage, with special regard to glial involvement. Aβ is a key molecular factor in the etiology of AD, primarily targeting synaptic function^19–21^, in a process that is rather slow over the years before massive synaptic loss and neuronal degeneration occur^22–24^. The events preceding full synaptic dysfunction, roughly coinciding with pre-symptomatic stages of disease, remain yet largely undiscovered but cover a crucial time span for preventive intervention^6,22^.

We previously demonstrated that OHC exposed to sub lethal concentrations of aggregated Aβ42, undergo a significant up-regulation of pre-synaptic vesicle component synaptophysin, the meaning of which was attributed to a compensatory but ultimately inefficient response to synaptic toxicity^16^. Our current aim was then to identify new targets in order to favor such compensatory events and prevent their ultimate failure. We here focused our interest on BDNF as a likely candidate, given its involvement in synaptic function and in neuronal-glial crosstalk, as seen also in AD^14,18,25,26^.

We confirmed that mature BDNF was significantly increased at time points in which SYP expression was also induced in OHC exposed to sub-lethal Aβ concentration. In agreement with our result, a biphasic pattern of expression has been shown for BDNF in patients, which seems to exactly retrace the expression of SYP, with an early increase in conditions of MCI, followed by a drop towards advanced stages of AD^13,27^. Notably, with the aid of fluorescently labeled Aβ42 we were able to distinguish a close physical relation of the peptide specifically with microglial cells in the CA1 area of OHC, especially dense where neuronal cell bodies are also localized. This observation is consistent with evidence showing that, in response to a toxic environment, microglial cells are activated and migrate to the site of the lesion following cues released by injured neurons^11,28^. More specifically, microglia here observed are reminiscent of a rod-type microglia, with an elongated, sausage-like shape, aligning along neurons^29^, already described in brain tissue from AD patients^30^. The organotypic model recapitulates to a high degree the cytoarchitecture of the hippocampus where all main players, i.e. neurons, microglia and astrocytes coexist giving rise to a complex signaling interplay. By separately culturing the component cell types, we were able to identify microglia as the significant source of increased mature BDNF in response to Aβ. This result is consistent with the literature showing microglial release of BDNF following exposure to different toxic stimuli^31–33^. Although others have shown Aβ-induced up-regulation of BDNF also by astrocytes^34^, the diverging results could be accounted for by the dissimilar protocols used in that study compared to ours.

Exploring the effects of microglial mature BDNF induction on neuronal response under toxic conditions, we showed that CM from Aβ-challenged pMG was indeed required to reproduce over-expression of SYP as seen in OHC, as well as for restoration of compromised synaptic vesicle recycling and enhanced neuroprotection. Importantly, at longer times of exposure to Aβ (5 d), CM from microglia still yielded significant neuroprotection (not shown). Unexpectedly, increased mature BDNF release by microglia was not directly responsible for SYP increase in neurons, which relied instead on autocrine BDNF signaling on microglia itself. In contrast, direct application of BDNF to neuronal-like cells was able to both prevent SYP loss and exert neuroprotection. This apparent discrepancy is likely due to the fact that exogenous BDNF only counteracts Aβ-induced SYP reduction, whereas the effect of CMA is not limited to preserve, but rather to increase SYP expression. The effects of BDNF on SH-SY5Y cells are known to be mediated by TrkB activation^35^. p75 receptor is also expressed in SH-SY5Y neuron-like cells^36^, is sensitive to different neurotrophins and can play a role in BDNF action^37^. However, p75 is preferentially activated by pro-BDNF^37^ and a detailed characterization of the involvement of different forms of BDNF and their receptors deserves a deep analysis that goes beyond the aim of the present study.

The hypothesis of TNF-α being the BDNF-induced autocrine mediator in CMA was ruled out by our results, although it was confirmed that Aβ stimulation increased the cytokine’s content in pMG. It is feasible to reason that TNF-α activates inflammatory pathways in neurons that counterbalance the positive action of BDNF. This would provide an explanation for the reduced trend of neuroprotection of CMA compared to CMC from resting pMG. In fact, CMC from resting microglia prevented SYP loss without causing its over-expression and ameliorated synaptic function by increasing the rate of synaptic vesicle recycling. The over-expression of SYP detected in neuronal-like cells exposed to Aβ + CMA could be connected to the accumulation of the protein in clumps, possibly indicative of a loss of function, as described for other neurodegenerative diseases^38^. Altogether, our data point to a beneficial role of resting microglia on neuronal survival, that is still retained following exposure to Aβ, despite the induced release of inflammatory signals (e.g. TNF-α) along with pro-survival ones (e.g. BDNF). Perfectly in line with our interpretation, where CMs from pMG sustain an adequately neurotrophic milieu yielding protection against synaptic damage, progression of cognitive impairment has been linked to an insufficiently neurotrophic environment^39^. Although in a recent work by Hong *et al*.^40^, microglia was shown as responsible for early synaptic loss in AD mouse model brains, SYP was not reduced in this model whereas post-synaptic sites were mainly affected.

The protective action we have here shown for microglia is mainly effected through up-regulation of synaptic proteins and rescue of synaptic function. As mentioned, increasing attention has been paid in recent years to the earliest events in AD development, in which initial increase of SYP expression is central, as shown in patients^41^ and recapitulated in AD animal models^42^. While it seems conclusive that such events represent a compensatory response to synaptotoxic signaling^39,43,44^, the debate regarding their real consequences is still open. On one hand there is evidence that increased SYP and synaptic activity leads to excess glutamate release causing exacerbation of excitotoxicity^45^. On the other hand, an overall inverse correlation has been shown between synaptic protein levels and clinical severity in AD^27^, with cases of elderly individuals presenting extensive pathology but normal cognition linked to sustained increase of SYP levels^43,46^. In addition, in the 3xTg-AD mouse model, increase in SYP neuronal content is still able to rescue some cognitive behaviors even after a prior decline in its levels^42^. Thus in this debate, our work adds to the evidence of a positive outcome linked to early SYP increase.

Our *in vitro* experimental settings allowed us to directly assess the interaction between microglia and neurons and the results here reported well correlate with *in vivo* studies where microglia-neuron cross-talk has been largely demonstrated in animal models of AD^47–49^. Interestingly, in this respect, although the real role of microglia in AD is still debated, recent data point to a subset of microglial population associated with restriction of AD development^50^.

In conclusion, we here provide substantial data in support of a beneficial contribution of microglia to the early compensatory response of neurons to Aβ. Microglia appears to strengthen neuronal resistance to synaptotoxicity, leading to enhanced neuronal viability, thus representing a compelling target for early intervention in AD.

## Materials and Methods

### Drugs and reagents

Aβ(1-42) peptide from Innovagen (Lund, Sweden) was dissolved in dimethylsulfoxide (DMSO) as a 5 mM stock, subsequently diluted to 100 μM in culture medium and enriched in oligomers by aggregation at RT for 24 h followed by at least 2 freeze-thaw cycles prior to use. Aβ(1-42) 5-FAM (FAM-Aβ) was dissolved in DMSO at an initial concentration of 200 μM. Human recombinant brain-derived neurotrophic factor (BDNF) and human recombinant tumor necrosis factor-α (TNF-α) were from PeproTech (London, UK). Pan-Trk inhibitor GNF5837 (Santa Cruz Biotechnologies; Santa Cruz, CA, USA), selective TrkB antagonist ANA-12 (Sigma-Aldrich Co, Milan, Italy), MAPK pathway inhibitor U0126 (Tocris Cookson Ltd, North Point, UK) were always added 10 min before other drugs.

### Organotypic hippocampal cultures (OHC)

OHC obtained from post-natal day 7 Sprague-Dawley rats (Harlan, Udine, Italy) were cultured with the interface method as previously described^51^, with slight modifications. Briefly, each hippocampus was isolated and cut into 350 μm-thick transversal sections with a McIlwain tissue chopper. Intact slices were selected and incubated for 20 minutes at 4 °C in Hank’s Balanced Salt Solution (Invitrogen Life Technologies, Milan, Italy) supplemented with 0.5% glucose (Sigma) and 1.5% Fungizone (Invitrogen). Slices were then transferred onto Millicell-CM culture inserts (MilliporeMerck KGaA, Darmstadt, Germany; 4 slices/insert) placed on top of 1 ml of Eagle’s minimal essential medium containing 25% HBSS, 25% horse serum (HS), 1 mM glutamine, 1.5% Fungizone (all from Invitrogen) and 0.5% glucose (Sigma). For experiments in serum-free conditions, HS was substituted with an equivalent volume of MEM. Cultures were incubated at 37 °C and 5% CO_2_ in a humidified atmosphere and subjected to medium change twice a week. Slices were used for experiments after 14 days of maturation *in vitro*.

### Cell cultures

Mixed glial cultures were prepared from 1–3 days-old Sprague-Dawley rats (Harlan). In brief, after removal of meninges and isolation of cortexes, cells were dispersed by mechanical and enzymatic dissociation with 0.25% trypsin (Invitrogen) and filtered through 40 μm nylon cell strainers (BD Biosciences, Erembodegem, Belgium). Cells were plated onto 75 cm^2^ flasks and maintained in DMEM supplemented with 10% fetal bovine serum (FBS), penicillin (100 U/ml)/streptomycin (100 µg/ml) at 37 °C and CO_2_ atmosphere. Confluent cultures at 8–10 d *in vitro* were shaken at 250 rpm and 37 °C for 1.5 h to collect microglia. Shaking was then protracted for 6 h at 500 rpm to remove oligodendrocytes, while remaining astrocytes were collected by trypsin digestion. For experiments, microglia and astrocytes were plated in 35 mm-dishes at a density of 3 million and 1.5 million cells, respectively. Mixed glial cultures were plated in 35 mm-dishes at a density of 1 million cells. All glial cells were used for experiments 48 h after plating. Isolated astrocyte and microglia cultures were up to 98% pure, as evaluated by flow cytometry with selective cell type markers. SH-SY5Y human neuroblastoma cells were cultured in DMEM/F12 supplemented with 10% FBS, penicillin (100 U/ml) and streptomycin, (100 μg/ml) at 37 °C and CO_2_ atmosphere. Based on experimental needs, cells were plated with the following densities: 600 k cells/well in 12-well plates, 30 k/well in 96-well plates and 100 k/well in 8-well microslides. Serum reduction was started the following day. All medium constituents were obtained from Invitrogen. Microslides were from Ibidi, all other plastic was from Falcon.

All animal experimental procedures were carried out in accordance with the directives of the Italian and European Union regulations for the care and use of experimental animals (DL116/92) and were approved by the Italian Ministry of Health.

### Western blot analysis

Cells were lysed in M-PER lysis buffer (Thermofisher Scientific, Waltham, MA, USA) supplemented with anti-protease cocktail mix (Sigma). Protein concentration was determined using micro Bradford reagent (Sigma) protocol according to the manufacturer’s instructions and measuring absorbance with a Varioskan^TM^ Flash Multimode Reader. SDS-PAGE was performed loading 20–50 μg of protein extracts on pre-cast 4–20% gradient gels (Bio-Rad, Hercules, CA, USA) and followed by transfer to nitrocellulose membrane (Hybond ECL, Amersham Biosciences Europe GmbH, Milan, Italy) using a Transblot semidry transfer cell (Bio-Rad). Membranes were blocked with Odyssey Blocking buffer and incubated with primary antibodies overnight at 4 °C. Primary antibodies used were: rabbit anti-BDNF (1:500; S. Cruz sc-546), mouse anti-SYP (1:10000; S. Cruz sc-17750), goat anti-TNFα (1:200; S. Cruz sc-1351); rabbit anti-β-actin (1:5000; Sigma A2066), and mouse anti-α-tubulin (1:10000; Sigma T9026). Membranes were then washed and exposed to appropriate IRDye^®^ 680RD or IRDye^®^ 800CW secondary antibodies (1:15000; LI-COR Biosciences, Lincoln, NE, USA) for 45 min RT. Detection of specific bands was carried out using the LI-COR Odyssey^®^ Infrared Imaging System. Band intensity was analyzed using the Image J software, developed by NIH and in public domain. All blots were cropped to display only specific bands of interest. Full length blots are available as Supplementary Information.

### Immunofluorescent staining

Fixation was carried out with ice-cold 4% paraformaldehyde (2 h for OHC; 30 min for cells), followed by permeabilization when necessary with 0.1% Triton X-100 on ice (1 h for OHC; 10 min for cells) and blocking in 3% BSA (2 h for OHC; 30 min for cells). Incubation with primary antibodies was carried out in a 3% BSA solution (24 h for OHC; overnight for cells) at 4 °C. Primary antibodies used were: chicken anti-integrin α-M (integrin; 1:80, Aves Labs MAC, Tigard, OR, USA), mouse anti-GFAP (1:300, Cell Signaling #3670, Danvers, MA, USA), rabbit anti-BDNF (1:100; S. Cruz), mouse anti-MAP2 (1:100; #05-346, Merck-Millipore); mouse anti-SYP (1:150; S. Cruz). After washing, cells were incubated with secondary antibodies RT (2 h for OHC; 45 min for cells), washed and mounted with regular or DAPI-containing mounting solution (both from Sigma). Secondary antibodies used were: PE-anti-chicken (1:200; S. Cruz sc-3730), Alexa-Fluor 546-anti-mouse (1:300; Invitrogen A10036), Alexa-Fluor 488-anti-rabbit (1:300; Invitrogen A11088). Digital images were captured with a Zeiss Observer.Z1 microscope equipped with the Apotome.2 acquisition system (Zeiss, Oberkochen, Germany).

### Flow cytometry

Cells were gently collected and fixed for 30 min with ice-cold 4% paraformaldehyde. When necessary, cells were permeabilized with 0.1% Triton X-100 for 10 min on ice. After blocking with 3% BSA for 30 min, cells were incubated with primary antibodies overnight at 4 °C, then washed prior to incubation with secondary antibodies for 45 min at RT. Primary antibodies used were: chicken anti-integrin α-M (1:500, Aves), mouse anti-GFAP (1:500, Cell Signaling), rabbit anti-BDNF (1:500; S. Cruz), goat anti-TNFα (1:250; S. Cruz), rabbit anti-Iba1 (1:300; Novus Biologicals, NBP2-19019). Antibodies were appropriately combined in multiple labeling experiments. Secondary antibodies used were: PE-anti-chicken (1:200; S. Cruz), FITC-anti-goat (1:200; S. Cruz sc-2024), Alexa-Fluor 647-anti-rabbit (1:500; Invitrogen A31573), PE-anti-mouse (1:300; S. Cruz sc-3738). Data was acquired on the Amnis FlowSight^®^ Imaging Flow Cytometer and analyzed with IDEAS^®^ Software (Millipore).

### BDNF Enzyme-linked Immunosorbent Assay (ELISA)

Levels of mature BDNF in conditioned medium were determined with the biosensis^®^ BDNF Rapid^TM^ ELISA kit (Biosensis Pty Ltd, Thebarton, SA, Australia), strictly following the manufacturer’s instructions. Absorbance at 450 nm was measured with a Varioskan^TM^ Flash Multimode Reader.

### FM 1–43 Dye Imaging

Experiments were carried out according to our previously published protocol^16^. Briefly, SH-SY5Y cells were washed in a modified Tyrode solution (TS; in mM: 150 NaCl, 4 KCl, 2 MgCl2, 10 glucose, 10 HEPES, and 2 CaCl2, pH 7.4), exposed to 10 μM FM 1–43 dye (Invitrogen) in the same buffer for 5 min to label membranes, then stimulated with high-potassium (90 mM KCl) TS (HKTS; containing equimolar substitution of KCl for NaCl) for 10 min to stimulate synaptic vesicle exocytosis. After HKTS removal, cells were subjected to a 5 min recovery in the presence of FM 1–43 (10 μM) to allow complete recycling of synaptic vesicles, then extensively washed with TS to remove excess dye. Finally, cells were stimulated with HKTS for 15 min to allow complete unloading of dye-labeled vesicles. All steps were carried out in the presence of (2,3-dihydroxy-6-nitro-7sulfamoyl-benzo[f]quinoxaline-2,3-dione (NBQX, 10 μM; Tocris) to prevent recurrent activity during stimulation. Images were captured at the end of wash (coinciding with complete loading) and at the end of unloading using a Zeiss Observer.Z1 microscope connected to a digital camera, using the same exposure settings for all images. Quantitative analysis of fluorescence intensity (as mean gray value) after loading and unloading were performed on 40X magnification images using the Image J software developed by NIH and freely available on the web.

### 3-[4,5-dimethylthiazol-2-yl]-2,5-diphenyltetrazoliumbromide (MTT) viability assay

SH-SY5Y cells were incubated with 1 mg/ml MTT substrate (Sigma) for 2 h at 37 °C. Dimethylsulfoxide was added to obtain cell lysis and solubilization of formazan resulting from MTT reduction by viable cells’ mitochondrial activity. Absorbance at 545 nm was then measured with a Varioskan^TM^ Flash Multimode Reader.

### Statistical analysis

All data were from at least three independent experiments, run at least in triplicate. Statistical analyses were performed by Student’s t-test or one-way ANOVA, followed by Neuman-Keuls test for significance with GraphPad Prism Software (GraphPad Software, San Diego, California USA). p < 0.05 was taken as the criterion for statistical significance.

## Electronic supplementary material

Supplementary Information
