# Supplementary material for: The contribution of microglia to early synaptic compensatory responses that precede β-amyloid-induced neuronal death
Source: Sci Rep. 2018 May 8;8:7297. doi: 10.1038/s41598-018-25453-1 (PMC5940848; doi:10.1038/s41598-018-25453-1)
Supplement: Supplementary file 1 — Supplementary Information [file 41598_2018_25453_MOESM1_ESM.pdf]

Supplementary Information

The contribution of microglia to early synaptic compensatory responses that precede β-amyloid-induced neuronal death

Sara Merlo, Simona Federica Spampinato, Martina Beneventano and Maria Angela Sortino*

Department of Biomedical and Biotechnological Sciences, University of Catania, Catania, Italy

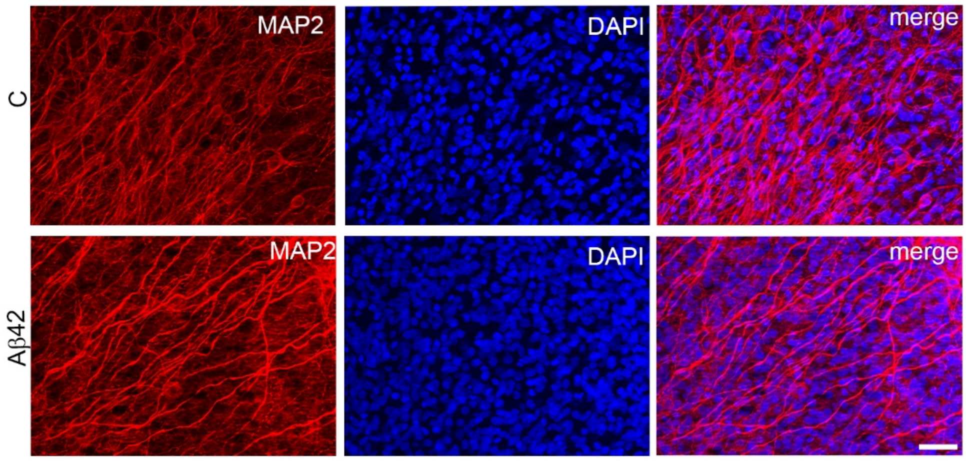

Supplementary Figure 1. MAP2 immunostaining in control or Aβ-treated OHC. OHC in serum free medium (C) or challenged with 0.5 μM Aβ42 for 24 h, were processed to label selective neuronal marker MAP2 (red). Nuclei were stained with DAPI (blue). Representative images are shown. Scale bar = 20 μm.

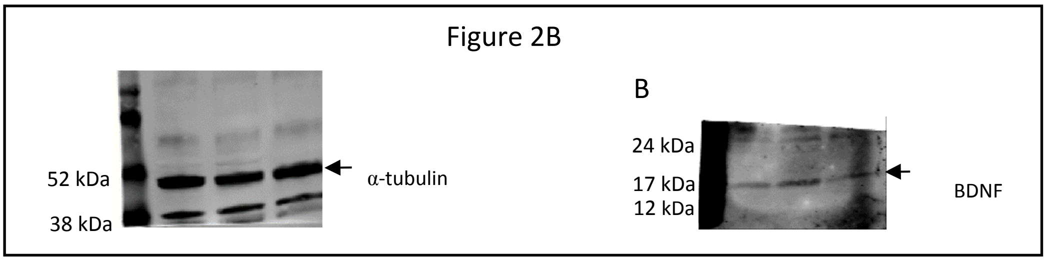

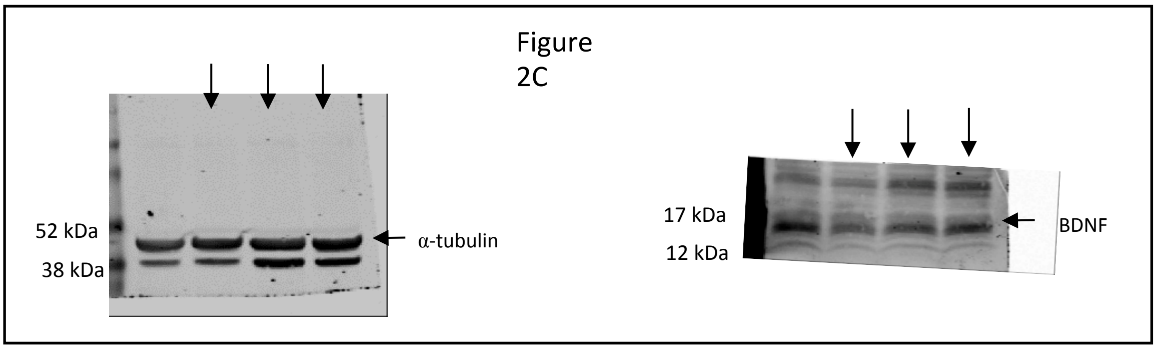

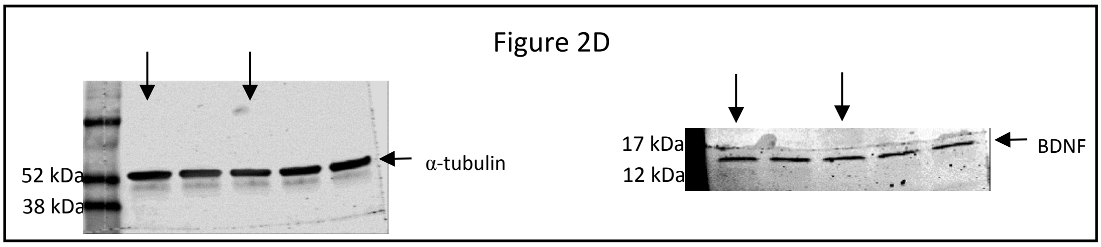

Supplementary Figure 2. Full lenght blots for α-tubulin and BDNF expression reported as cropped bands in Fig. 2 in the manuscript. Horizontal arrows indicate specific bands of interest and vertical arrows indicate specific lanes presented in final crops in Fig. 2. Selective regions of interest (ROI) in the blots were scanned during acquisition.

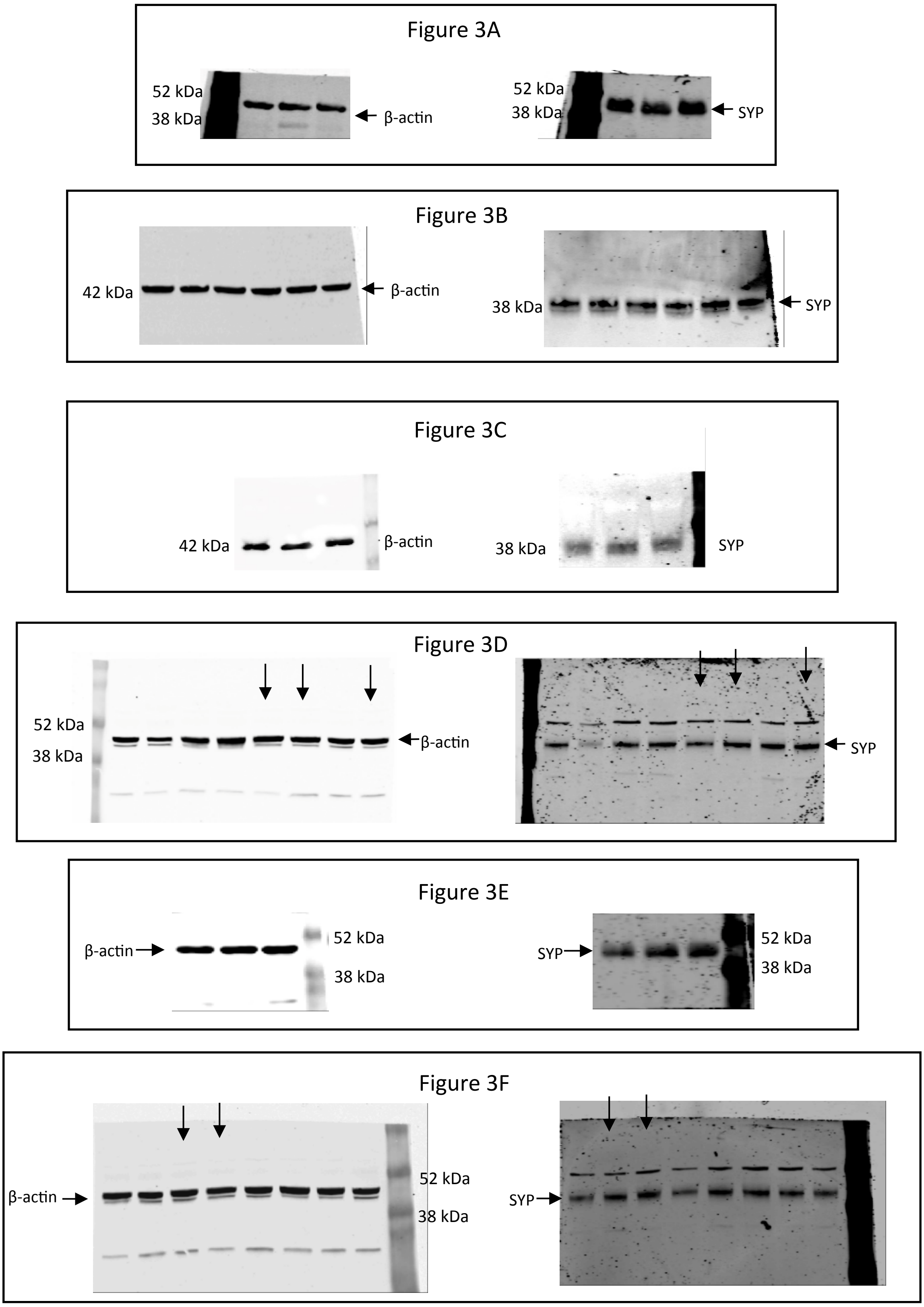

Supplementary Figure 3. Full length blots for β-actin and SYP expression in SH-SY5Y cells, reported as cropped bands in Fig. 3 of the manuscript. Horizontal arrows indicate specific bands of interest and vertical arrows indicate specific lanes presented in final crops in Fig. 3. Selective ROIs in the blots were scanned during acquisition.

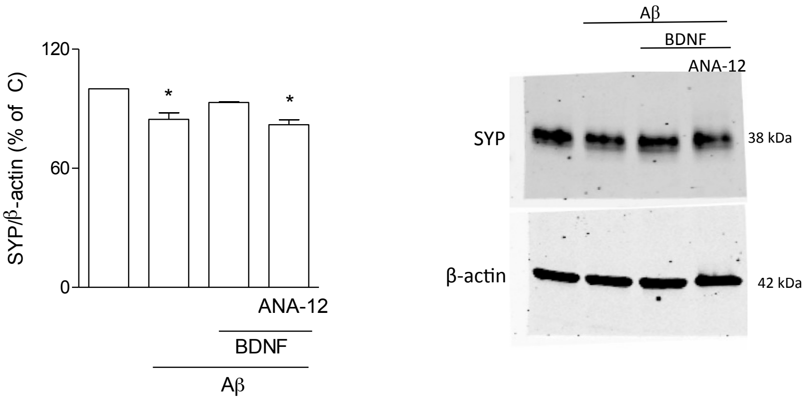

Supplementary Figure 4. The TrkB-selective inhibitor ANA-12 is effective in preventing BDNF action on SYP expression. Pre-treatment of SH-SY5Y cells with the selective TrkB inhibitor ANA-12 (20 µM; 10 min) prevented BDNF (1 ng/ml) effects on Aβ42-induced (0.5 µM; 5 h) SYP reduction. Bars are mean ± SEM of three independent experiments. $p<0.05$ vs. respective condition in the absence of Aβ (bar 2) or in the absence of ANA-12 (bar 4) by one-way ANOVA followed by Newman-Keul test for significance. A representative full length blot is shown.

Figure 4C

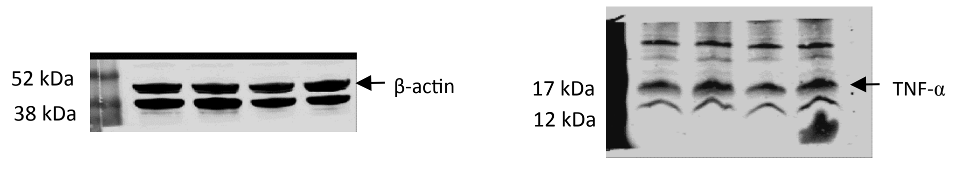

Figure 4D

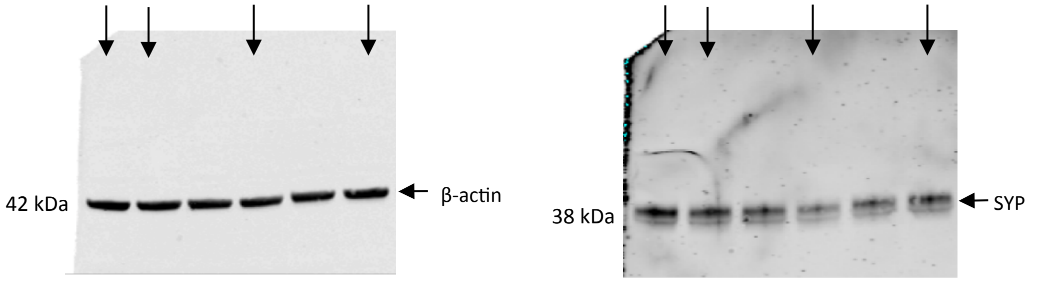

Supplementary figure 5. Full length blots for β-actin and TNF-α expression in pMG, and β-actin and SYP expression in SH-SY5Y cells, reported as cropped bands in Fig. 4 in the manuscript. Horizontal arrows indicate specific bands of interest and vertical arrows indicate specific lanes presented in final crops in Fig. 4. Selective ROIs in the blots were scanned during acquisition.
